# Efficacy and safety of modified Xiao-Feng Powder in the treatment of chronic urticaria: protocol of a randomized double-blind placebo-controlled study

**DOI:** 10.1186/s13020-022-00642-3

**Published:** 2022-07-22

**Authors:** Hing Yu Hung, Tianhe Song, Steven King Fan Loo, Kam Leung Chan, Jessica Yuet Ling Ching, Chi Him Sum, Louis Cho Wing Lo, Sarah Chon Pin Chia, Ray Tin Muk Ho, Pui Kuan Cheong, Tony Hon Chung Siu, Ka Chun Leung, Zhi-Xiu Lin

**Affiliations:** 1 Hong Kong Institute of Integrative Medicine, Faculty of Medicine, The Chinese University of Hong Kong, Shatin, N.T., Hong Kong SAR China; 2 School of Chinese Medicine, Faculty of Medicine, The Chinese University of Hong Kong, Shatin, N.T., Hong Kong SAR China; 3 Department of Medicine and Therapeutics, Faculty of Medicine, The Chinese University of Hong Kong, Shatin, N.T., Hong Kong SAR China

**Keywords:** Urticaria, Chinese medicine, Xiao-Feng Powder

## Abstract

**Background:**

Chronic Urticaria (CU), a common skin disorder known as Yin Zhen in Chinese medicine, is characterized by recurrent, pruritic, pink-to-red edematous lesions and wheals on the skin. Xiao-Feng Powder (XFP, meaning Wind-Dispersing Powder), is reported to be one of the most frequently used Chinese herbal formulae for CU. In this study, we aim to investigate the effectiveness and safety of modified Xiao-Feng Powder (mXFP) for the treatment of CU.

**Methods:**

In this randomised double-blind placebo-controlled clinical trial, 58 subjects identified as having mild to severe urticaria (Urticaria activity score greater than 10) will be recruited and randomised into two groups to receive antihistamine Bilastine with either mXFP or placebo for 12 weeks, followed by post treatment visits at week 16. The primary outcome measure is the change of weekly urticaria activity score (UAS7) at week 12. Secondary outcome measures include the Urticaria Control Test (UCT), Visual Analog Scale of Itch Severity (VAS), Chronic Urticaria Quality of Life Questionnaire (CU-Q2oL), Angioedema Activity Score (AAS), immunoglobulin E (IgE) test, gut microbiota test and use of antihistamines during study period. The trial will be conducted at three Chinese medicine clinics in Hong Kong.

**Expected outcomes:**

The results of this study will establish robust clinical evidence about the efficacy and safety of mXFP in the treatment of CU. A specific feature of this trial is that it is a integrative medicine trial with subjects being allowed to take the Western and Chinese medicine together for the treatment.

*Trial registration* This is registered on ClinicalTrials.gov, ID: NCT04967092. Register date: July 19, 2021. https://clinicaltrials.gov/ct2/show/NCT04967092.

## Background

Urticaria is a common skin disorder and 15–25% of people experience at least one attack of urticaria during their lifetime [1, 2]. It is characterized by recurrent, pruritic (itchy), pink-to-red edematous (swollen) lesions which usually have pale centres (wheals) [3]. The size of the lesions can range from a few millimetres to several centimetres in diameter [4, 5]. Approximately 40% of patients with urticaria also have angioedema (swelling that occurs beneath the skin) [5].

Urticaria can be divided into acute urticaria (AU) and chronic urticaria (CU) according to the disease duration. For CU, it is more common in adults, and affects women more frequently than men [5–7]. The prevalence of CU is estimated to be anywhere from 0.5 to 5% in the general population [8]. Moreover, CU can be classified as either chronic autoimmune urticaria or chronic idiopathic urticaria. In chronic autoimmune urticaria, circulating immunoglobulin G (IgG) autoantibodies react to the alpha subunit of the high-affinity IgE receptor on the dermal mast cells and basophils, leading to chronic stimulation of these cells and the release of histamine and other inflammatory mediators, thereby causing urticaria and angioedema [9]. Patients with chronic idiopathic urticaria do not have evidence of autoimmunity. In this form of urticaria, there appears to be persistent activation of mast cells, but the mechanism of mast cell triggering is unknown.

In Chinese medicine (CM), CU is known as Yin Zhen [10, 11]. Urticaria is usually associated with either internal or external wind pathogen. Wind as a pathogenic factor is characterised by sudden onset and rapid changes in symptoms, and when resulting from an internal deficiency, it often leads to dryness. A constitutional weakness, inherited from conception and/or during pregnancy, may predispose a person to CU. CU can also be caused by deficiency of Qi and blood or failure of defensive Qi in protecting the exterior. When deficiency of defensive Qi is prolonged, the patient will become more susceptible to repeated external wind attacks (CU). Persistent wind pathogen attack causes the skin dryness, leading to the development of wheals and pruritus [11–13].

In 2012, the China State Administration of Chinese Medicine issued the “Guidelines for Diagnosis and Treatment of Common Diseases of Dermatology in Traditional Chinese Medicine” [14]. Syndrome differentiation is an important feature of diagnosis in CM practice and the main syndrome types of Yin Zhen include the “Syndrome of wind-heat attacking the exterior (exterior heat syndrome)”, the “Syndrome of blockage of the exterior by wind and cold (exterior cold syndrome)”, and the “Syndrome of blood deficiency and wind-dryness” [13–15]. In addition to syndromes described above, four additional syndrome types were also described in this guideline. These include the “Damp-heat in the stomach and intestine”, the “Defence-exterior insecurity” (defensive Qi deficiency), the “Deficiency of Qi and blood”, and the “Qi and blood stagnation and stasis” [14]. The treatment strategies of CU usually aim for correcting respective aetiopathogenesis, therefore include such methods as “clearing heat and dispelling wind”, “dispelling wind and stopping itchiness”, “tonifying Qi and securing the exterior”, and “tonifying yin and moistening dryness”.

Nowadays, CM is widely used for managing CU in mainland China and Hong Kong [16, 17]. Several research groups have conducted clinical studies to evaluate the efficacy of Chinese herbal medicine for the treatment of CU in recent decades [18]. Among different Chinese herbal formulae for urticaria, Xiao-Feng Powder (XFP), literally meaning Wind-Dispersing Powder, is one of the most frequently prescribed Chinese herbal formulae for CU [19, 20].

XFP is a famous Chinese herbal formula that first appeared in the “Orthodox Lineage of External Medicine” written in 1617. It consists of *Angelicae Sinensis Radix* (Dang-Gui), *Rehmanniae Radix* (Sheng-Di-Huang), *Saposhnikoviae Radix* (Fang-Feng), *Cicadae Periostracum* (Chan-Tui), *Anemarrhenae Rhizoma* (Zhi-Mu), *Sophorae Flavescentis Radix* (Ku-Shen), *Sesami Nigrum Semen* (Hu-Ma), *Schizonepetae Herba* (Jing-Jie), *Atractylodis Rhizoma* (Cang-Zhu), *Arctii Fructus* (Niu-Bang-Zi), *Gypsum Fibrosum* (Shi-Gao), *Glycyrrhizae Radix et Rhizoma* (Gan-Cao) and *Akebiae Caulis* (Mu-Tong). XFP has been widely used in Chinese medicine dermatology to expel wind and eliminate dampness, clear heat, nourish the blood and stop itchiness [21]. Moreover, other researchers have found that XFP is effective in inhibiting inflammation, allergy, and oxidative stress in allergic skin diseases [22]. This formula is applicable for urticaria due to wind, dampness, and heat invading the body and settling in the blood vessels where it is unable to vent externally or drain internally. The symptoms that can be managed by XFP include itchy skin, red rashes and weeping upon scratching. This formula serves as the common formula for urticaria and eczema caused by wind-heat or wind-dampness trapped between the skin, striae, and interstices.

This clinical study aims to determine the effectiveness and safety of modified Xiao-Feng Powder (mXFP) for the treatment of CU. A randomised, double-blind, placebo-controlled clinical trial design with strong scientific rigor will be employed in this study. In the study, we will use comprehensive outcome measures to evaluate the treatment response. Furthermore, we will perform gut microbiome analysis to explore the relationship between the treatment effect of mXFP and the modulation of gut microbes. By collecting subject’s fecal samples, the richness of 16S rDNA and the biodiversity in each gut microbiome sample can be compared before and after taking mXFP [23]. This clinical study will be able to provide robust clinical evidence on the efficacy and safety of mXFP for CU.

### Objectives

The objective of the study is to evaluate the clinical efficacy and safety of mXFP on patients with CU.

## Methods

### Study design

It is a multi-centred, randomized double-blinded placebo-controlled clinical trial with treatment duration of 12 weeks and post-treatment follow-up period of 4 weeks. We will randomly allocate the eligible subjects into two groups to receive either mXFP or placebo twice daily for 12 weeks followed by post treatment visits at week 16. Subjects will also take antihistamine standard therapy regularly for the first 6 weeks, and then for on-demand basis for the next 6 weeks during the treatment period.

### Inclusion criteria

Men and women aged from 18 to 65 years with mild to serve chronic urticaria, who can provide informed consent for the participation, complete questionnaires and take medications as scheduled, are included. Chronic urticaria with mild to serve activity is defined by the following criteria:

1. Documented history of CU for at least 6 weeks prior to entry in the study;
2. Meet EAACI/GA^2^LEN/EDF/WAO 2017 guidelines, i.e. spontaneous appearance of wheals, angioedema or both for > 6 weeks due to known or unknown causes; and
3. Symptom severity must be greater than 10 points (UAS7 score).

### Exclusion criteria

Subjects are excluded if they meet the any one of the following criteria:

1. Dual deficiency of Qi and blood according to Chinese medicine theory
2. Urticaria is induced by physical factors (e.g., cold urticaria, delayed pressure urticaria, solar urticaria, heat urticaria, vibratory angioedema) only;
3. Known to have dermatological diseases with skin pruritus;
4. Known to have any serious diseases such as cancer, severe kidney and liver impairments, autoimmune disease, thyroid disease, Hodgkin’s disease, lymphoma, severe mental disorders, leukemia, and acute infectious disease;
5. Known to use oral/injectable corticosteroids, leukotriene inhibitors, immunosuppressants or other Chinese herbal medicine within one month of enrollment;
6. Known to receive omalizumab, ligelizumab, or other experimental biologic for CU;
7. Documented pregnancy or planning to conceive, breast-feeding women; or
8. Operate heavy machinery or need to drive motor vehicles as an essential part of their profession.
9. Known to recent history (within previous 12 months) of drug addiction or alcohol abuse.
10. Involved in other interventional clinical studies at the same time.
11. Use antibiotics, prebiotics and probiotics within 4 weeks before study treatment commencement.

### Outcomes

The primary outcome measure of this trial is the change of weekly urticaria activity score (UAS7) at week 12. There are multiple secondary outcomes, including the change of UAS7 score at week 6 and 16, the change of Urticaria Control Test (UCT), Visual Analog Scale of Itch Severity (VAS), Chronic Urticaria Quality of Life Questionnaire (CU-Q2oL) and Angioedema Activity Score (AAS), at week 6, 12 and 16, the change of immunoglobulin E (IgE) test and gut microbiota test at week 12, and the use of the standard antihistamine therapy (Bilastine) during the entire study period.

### Intervention

In this trial, the dose of standard therapy of antihistamine, Bilastine, is 20 mg once daily during the first 6 weeks of treatment, and on-demand basis during 6th-12th weeks. Participants will be allowed to increase dose of Bilastine up to 20 mg stat dose in case of insufficient control of symptoms according to EAACI/GA2LEN/EDF/WAO 2017 guidelines [3]. The dose of the investigational Chinese medicine formula granules, mXFP, is 19.4 g each time, twice daily in the treatment group while the placebo granules is 19.4 g each time, twice daily in the control group. All participants will be randomly assigned at a 1:1 ratio into two groups to receive the granules.

Moisturiser is not prohibited for use during the study. However, any additional treatment other than the standard western therapy and Chinese medicine granules after randomization is prohibited. Besides, participants will not be allowed to take prebiotics and probiotics during the study.

### Safety

No documented severe or serious event was reported using this herbal formula [23, 24]. However, there are still minor adverse events such as abdominal pain and dyspepsia. [25]. Safety will be assessed by laboratory parameters and participants’ reported adverse events and/or serious adverse events. All participants will be suggested to inform the study team about any adverse reaction during the treatment and follow-up periods. A direct telephone line will be provided so that subjects can report any adverse events during office hours between scheduled visits. Participants will be arranged to see our doctors if needed as unscheduled visits. All adverse reaction will be recorded, and the treatment suspended when any severe adverse reaction occurs.

Besides, in order to ensure participants’ safety, general data and routine blood test will be done before taking the medications. Blood test may induce bleeding, infection, bruising, and feeling lightheaded. When the needle pricks participant’s arm or hand, the participant may suffer a mild painful feeling, and the site maybe sore afterward. However, the risk of blood test is very low and discomfort is mild.

### Recruitment

CU participants will be recruited from the following Chinese medicine clinics: (1) The CUHK Chinese Medicine Specialty Clinic and Teaching and Research Centre on CUHK campus (CUHK-CMSCTRC); (2) The two Integrative Medical Centres, Hong Kong Institute of Integrative Medicine at Sha Tin and Wan Chai. Advertisements in the poster on the clinic and internet platforms, such as Facebook, emails, and website will be made to facilitate community recruitment. Besides, we will publish articles in local newspapers and magazines as well as organize health promotion talks to augment the patient recruitment process. Potential participants with CU who meet the eligibility criteria will be recruited.

### Randomisation and blinding

A random number table will be generated with a computerized random number generator program. The random allocations will be put into opaque envelops with sequential study numbers. Two sets of the envelope will be prepared, with one set for randomization at the site and another set stored in the investigator’s office for emergency unblinding. Each participant will be assigned with a sequential study number and then the corresponding allocated herbal granules or placebo will be prepared according to the random allocation sequence by an independent research staff member. The dosages will be prepared according to CMP’s instruction. During the study, the CMP investigators, study participants and outcome assessors will be kept blind of the allocated intervention.

### Trial procedures and follow-up

**Fig. 1:**
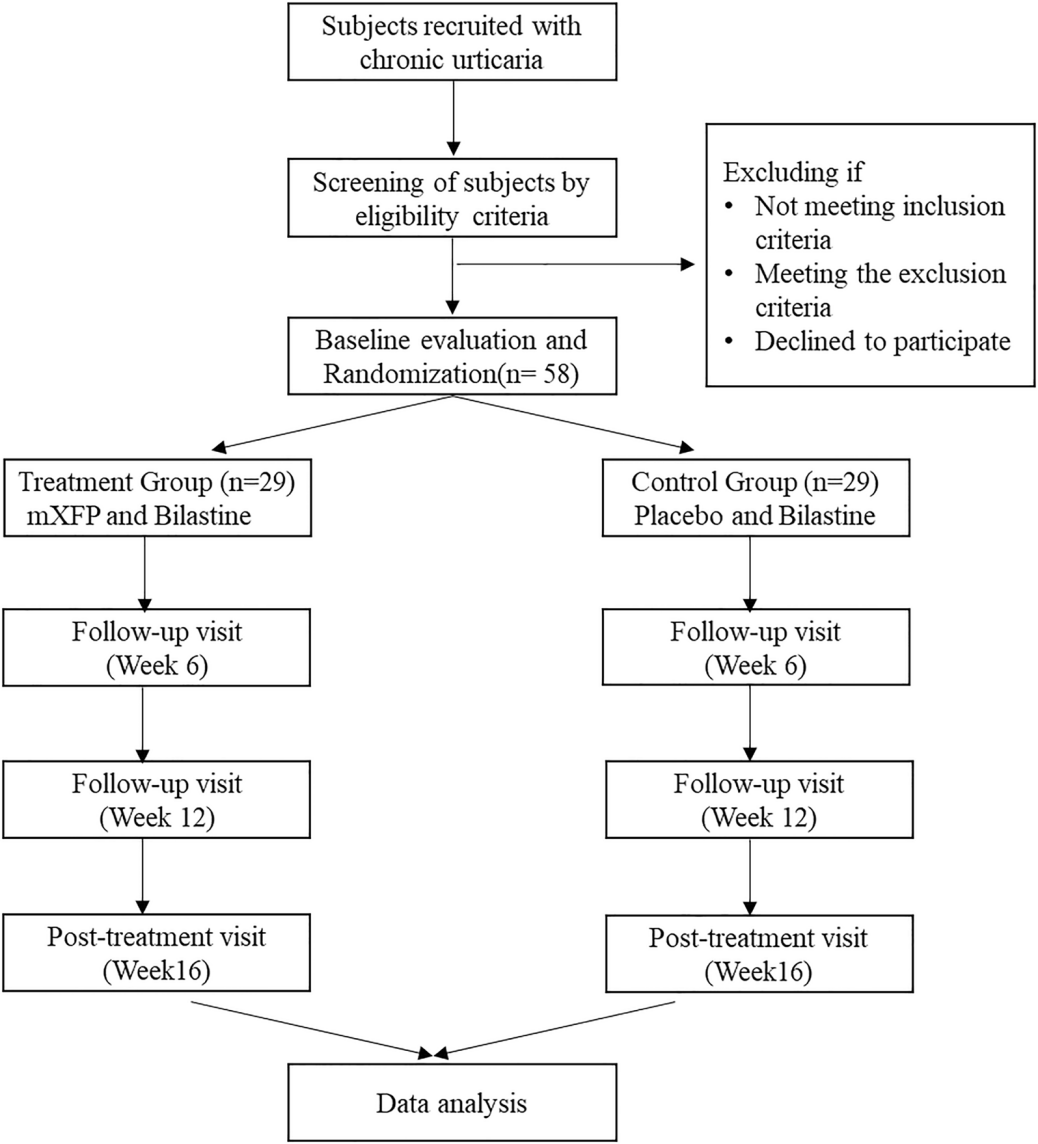
Study flow chart

As shown in the Fig. 1 and Table 1, each partcipant has a phone call or face-to-face interview in screening and a total four visits to the Chinese medicine clinic throughout the study. Community participants who express interest in our study and agree for our telephone screening will leave their contact to project research assistant while potential participants referred from the wards, clinics or medical centres will be screened directly. Eligible subjects after screening will be arranged for a face-to-face or telephone interview for eligibility assessment. UAS7 will be delivered and filled by participants 7 days before the baseline visit.

**Table 1:** SPIRIT schedule of enrolment, interventions, and assessments

	Study period				
	Screening	Treatment period			Post treatment follow up
Timepoint	−7 to 0 days	Day 0 (baseline)	Week 6 (±4 days)	Week 12 (±4 days)	Week 16 (±4 days)
Enrolment					
Informed consent	X				
Eligibility screen	X	X			
Medical consultation and assessment	X	X	X	X	X
Medical history	X				
Concomitant medication	X	X	X	X	X
Intervention					
mXFP/ Placebo and Bilastine		X	X		
Outcome Assessment					
Weekly urticaria activity score (UAS7)	X	X	X	X	X
Urticaria Control Test (UCT)		X	X	X	X
Visual Analog Scale of Itch Severity (VAS)		X	X	X	X
Chronic Urticaria Quality of Life Questionnaire (CU-Q2oL)		X	X	X	X
Angioedema Activity Score (AAS)		X	X	X	X
Serum levels of IgE	X			X	
Gut microbiota		X		X	
AE/SAE assessment			X	X	X
Laboratory Assessment					
CBP with differentiation	X			X	
Renal and Liver function test	X			X	
Thyroid function test (FT3, FT4 and TSH)	X				

For the baseline visit, eligibility will be checked and information about the study will be explained to the participants who will sign informed consent form voluntarily at their agreement afterward. Participants will be taken for not more than 20 ml of blood for complete blood count (CBC), liver function test (LFT), renal function test (RFT), and thyroid function test (FT3, FT4 and TSH) as safety measures while total immunoglobin E (IgE) as one of the outcomes for the measurement of inflammation. Then participants will be randomly assigned at a 1:1 ratio to receive 6 weeks of either mXFP granules 19.4 g each time or placebo granules 19.4 g each time twice daily. All participants will take the standard therapy of Bilastine 20 mg once daily during the first 6 weeks of treatment, and on-demand basis for the subsequent 6 weeks. They will be allowed to increase the dose of oral Bilastine up to 20 mg stat dose in case of insufficient control of symptoms according to EAACI/GA2LEN/EDF/WAO 2017 guidelines [3]. Baseline demographic data, medical history and drugs including use of moisturiser will be taken. UAS7, UCT, VAS, CU-Q2oL, and AAS will be completed. Participants will start study treatment after stool collection and notification of non-clinically significant blood results for the safety measures. Relevant information on the use and storage of study drugs will be recorded.

Participants will return for follow-up at week 6 (± 4 days), week 12 (± 4 days), and be followed by a post-treatment visit at week 16 (± 4 days). UAS7, UCT, VAS, CU-Q2oL, and AAS will be collected at every follow-up visits. At the 3rd follow-up visit at week 12, stool samples and blood samples for CBC, LFT, RFT and total IgE will be collected. Adverse events will be recorded for safety monitoring.

Throughout the study, participants are required to keep a daily record for their compliance and side effects of the study treatment, if any. Also, the participants are advised to record all the other medication, including health food supplements during the period of study. For the stool sample, as the food intake may affect the gut species and influence the gut microbiome analysis result, participants are required to record three days’ food diary before the day of taking faecal specimen.

### Sample size

The primary outcome is the UAS7 after 12 weeks of treatment. The minimal clinically important difference of UAS7 for measuring chronic urticaria ranges from 9.5 to 10.5 [26–28]. The standard deviation was estimated to be 12 from a previous study about the treatment of Chinese herbal formulae including Xiao-Feng Powder on Chinese patients with CU [24]. To detect a clinically meaningful difference of 10 in UAS7, with 80% power at the 0.05 significance level and an expected standard deviation of 12, 24 cases in each group are needed. To account for potential dropouts, a total of 58 subjects will be needed. PASS 12.0 was used for the sample size calculation.

### Statistical analysis plan

All analyses will be conducted according to the intention-to-treat principle. Descriptive statistics will be computed for each of the analysed variables. The primary efficacy analysis will be done by comparing the two groups with respect to the UAS7 at baseline and at 6, 12 and 16 weeks after randomization using analysis of covariance (ANCOVA). Supplementary analyses will include a complete-case analysis and a linear mixed model analysis of all available data. Repeated measures ANCOVA will be used to test for group differences in the secondary outcomes, with adjustment for relevant baseline covariates. All statistical tests will be two-sided, and *p* < 0.05 is considered significant difference. The statistical software of SPSS 23.0 will be used for analysis.

Adverse events will be categorized, and the percentage of those experiencing mild and serious adverse events will be documented. Chi-square tests will be performed to examine differences in the proportion of total and categories of adverse events within each group.

### Data management and quality assurance

To protect patient privacy, all research data will be handled in line with Hospital Authority and Hospital’s policy in handling, storage and destruction of participants’ medical records. The data would be locked in cabinets where the department keeps the participants’ confidential information. Besides, all collected information will be input into a computer with restricted access to investigators only. Only de-identified data will be collected and removed from the premises. Participant information will be kept in storage for 7 years after the study and will be destroyed afterwards. 

### Ethical consideration and dissemination of information

This study will be conducted according to the Declaration of Helsinki and ICH-GCP. The ethical validity of the study was approved by the Joint The Chinese University of Hong Kong–New Territories East Cluster Clinical Research Ethics Review Committee (CREC Ref. No. 2021.085 T). Any modifications to the protocol will be reported to this committee, and amendment approval should be obtained before any changes can take place. Participants will be informed that the confidentiality of all information and data will be maintained anonymity. Consent forms should be signed by the participants before the study. All information will be encrypted and only the involved investigators can have access. Password is required to access the data. Participants are free to withdraw at any time without giving a reason or punishment.

## Discussion

Xiao-Feng Powder (XFP) is a widely used Chinese medicine formula for managing chronic urticaria (CU) in Chinese medicine and thus there is widespread interest in the potential role of modified Xiao-Feng Powder (mXFP) in the CU treatment. This prospective randomized double-blinded placebo-controlled clinical study allow evaluating the clinical efficacy and safety of mXFP in the treatment of CU in different aspects. It can help clarify the role of mXFP in future CU treatment. The study has several strengths: diverse advertisement and multi-centred recruitment enable enrolment of subjects from different groups, several clinically relevant follow-up visits enable the researchers to monitor the subjects’ body condition, double blind design minimizes subjective biases, and diverse study assessments enable comprehensive data analysis.

Frist of all, CU participants are recruited from three clinics which located on CUHK campus, and in Sha Tin and Wan Chai districts. Advertisements in the poster on the clinic and internet platforms, articles in local newspapers and magazines as well as health promotion talks will also be made to facilitate community recruitment. These enable more potential participants with CU who meet the eligibility criteria be recruited, and thus minimize the selection bias and ensure that the sample is representative of the CU population. Second, medical consultation will be arranged for each follow-up visit. Chinese medicine practitioners will assess participants’ body condition and participants are also required to record all medication intake and the side effects. These arrangements enable the researchers to monitor the body condition and the compliance of subjects during the treatment. Third, participants, clinicians and researchers are all blinded during the study. None of them would know if the participants were receiving mXFP or placebo until the study is completed. It can minimize the placebo effects, observer effect and bias. Fourth, efficacy of mXFP in different aspects can be evaluated by comprehensive study assessments. It can provide a full picture of information about the role of mXFP in CU treatment.

Nevertheless, a number of limitations require further comment. First, according to Chinese medicine theory, treatment is based on syndrome differentiation but not disease. mXFP is used to expel wind and eliminate dampness, clear heat, nourish the blood and stop itchiness, but not for deficiency of Qi and blood. Therefore, it is not suitable for all chronic urticaria subjects. Subjects are excluded if they have dual deficiency of Qi and blood. To enhance the reliability of inclusion, CMP will have detailed consultation with the subjects in screening. Moreover, Chinese medicine consultation will be held at every visit to keep monitoring the body condition. Second, as participants would intake both Western and Chinese medicine in the treatment, it may be difficult to show the causation between the efficacy of mXFP and urticaria treatment effect. However, the administration of oral mXFP and Bilastine adopts a dose regime similar to that currently used in daily practice. Although it may not directly reflect the causation, the correlation between the mXFP treatments and outcomes in real-world health system practice can be observed in the study. Moreover, the primary efficacy analysis will be done by comparing the two groups, the treatment and control group. Descriptive statistics will be computed for each of the analysed variables.

## Conclusion

In conclusion, this protocol will provide details for investigators about the mXFP in treating chronic urticaria. The results of this study would be able to contribute robust evidence concerning whether mXFP is effective and safe in the treatment of chronic urticaria. The results may also help to discover the underlying mechanism of this herbal remedy for chronic urticaria.

## Data Availability

The datasets used and/or analysed during the current study are available from the corresponding author on reasonable request.

## Abbreviations

AAS: Angioedema Activity Score
ANCOVA: Analysis of covariance
AU: Acute urticarial
CBC: Complete blood count
CM: Chinese medicine
CMP: Chinese Medicine Practitioner
CREC: Clinical Research Ethics Review Committee
CU: Chronic urticaria
CUHK: The Chinese University of Hong Kong
CU-Q2oL: Chronic Urticaria Quality of Life Questionnaire
EAACI: The European Academy of Allergology and Clinical Immunology
EDF: The European Dermatology Forum
FT3: Free triiodothyroinine
FT4: Free throxine
GA^2^LEN: The EU-founded network of excellence, the Global Allergy and Asthma European Network
ICH-GCP: International Council on Harmonisation of Technical Requirements for Registration of Pharmaceuticals for Human Use- Good clinical practice
IgE: Immunoglobulin E
IgG: Immunoglobulin G
LFT: Liver function test
mXFP: Modified Xiao-Feng Powder
RFT: Renal function test
SPSS: Statistical Product and Service Solutions
TSH: Thyrotropin
UAS7: Weekly urticaria activity score
UCT: Urticaria Control Test
VAS: Visual Analog Scale of Itch Severity
WAO: The World Allergy Organization
XFP: Xiao-Feng Powder
